# Design and Validation of a 150 MHz HFFQCM Sensor for Bio-Sensing Applications

**DOI:** 10.3390/s17092057

**Published:** 2017-09-08

**Authors:** Román Fernández, Pablo García, María García, José V. García, Yolanda Jiménez, Antonio Arnau

**Affiliations:** 1 Advanced Wave Sensors S. L., Algepser 24, 46988 Paterna, Spain; pgarcia@awsensors.com (P.G.); mgarcia@awsensors.com (M.G.); jvgarcia@awsensors.com (J.V.G.); 2 Centro de Investigación e Innovación en Bioingeniería, Universitat Politècnica de València, Camí de Vera S/N, 46022 Valencia, Spain; yojiji@eln.upv.es (Y.J.); aarnau@eln.upv.es (A.A.)

**Keywords:** HFF-QCM (high fundamental frequency quartz crystal microbalance), finite element method (FEM), flow cell, biosensor, PoC (point of care), MQCM (monolithic quartz crystal microbalance)

## Abstract

Acoustic wave resonators have become suitable devices for a broad range of sensing applications due to their sensitivity, low cost, and integration capability, which are all factors that meet the requirements for the resonators to be used as sensing elements for portable point of care (PoC) platforms. In this work, the design, characterization, and validation of a 150 MHz high fundamental frequency quartz crystal microbalance (HFF-QCM) sensor for bio-sensing applications are introduced. Finite element method (FEM) simulations of the proposed design are in good agreement with the electrical characterization of the manufactured resonators. The sensor is also validated for bio-sensing applications. For this purpose, a specific sensor cell was designed and manufactured that addresses the critical requirements associated with this type of sensor and application. Due to the small sensing area and the sensor’s fragility, these requirements include a low-volume flow chamber in the nanoliter range, and a system approach that provides the appropriate pressure control for assuring liquid confinement while maintaining the integrity of the sensor with a good base line stability and easy sensor replacement. The sensor characteristics make it suitable for consideration as the elemental part of a sensor matrix in a multichannel platform for point of care applications.

## 1. Introduction

Point of care (PoC) devices are very promising diagnostic tools that can operate in different environments, from clinical labs to small-size hospitals or even at patient’s house, due to their unique characteristics. PoC platforms are designed to provide fast assay results, be portable and compact, and operate in a simple and automatic way. Such systems can be operated by non-specialists and provide valuable information about a large variety of diseases, e.g., cancer, cardiovascular, and infectious diseases [1].

The widespread application of PoC devices in common clinic practice will increase the prospects for more efficient patient monitoring during treatment, or even make available early diagnostic mechanisms for disease prevention in high-risk populations.

PoC platforms are typically based on genomics and/or proteomics for the detection stage, and require low cost, fast, sensitive, reliable and small-size transduction technologies in order to be successfully implemented [2].

Acoustic wave sensors are perfectly suited to fulfill these requirements. Health care, food security, and environmental monitoring fields are all market applications in which acoustic sensing has shown its potential. Acoustic sensing has taken advantage of the progress made in the last decades in piezoelectric resonators for radio frequency (RF) telecommunication technologies. The piezoelectric elements used in radars, cellular phones, or electronic watches for the implementation of filters, oscillators, etc., have been applied to these types of sensors.

Among the different approaches for the design of acoustic wave sensors, QCM (quartz crystal microbalance) is probably the most used sensing technology. Its operation is based on the so-called gravimetric technique [3], which relates resonance frequency shifts of the resonator to mass changes on the sensor surface. It has opened a great deal of applications in bio-chemical sensing: immunoassays, protein adsorption, DNA hybridization, etc. [4,5]. The main advantages of the QCM technique include: direct label-free detection, real-time non-invasive approach, low cost, and the ability to detect viscoelastic and conformational changes of the physical layer close to the sensor surface [6]. In the QCM technique, selectivity is provided by the appropriate sensor surface functionalization.

Although QCM techniques have improved in robustness and reliability, and allow measuring molecular interactions in real time, the improvement of the sensitivity and the possibility of multi-analysis and integration capabilities can be considered remaining challenges. These characteristics are highly demanded in the field of point of care diagnosis.

MQCM (Monolithic QCM) devices [7] have emerged as very promising acoustic wave sensors that could overcome the current limitations of the QCM technique in bio-sensing applications. They consist of the integration of many QCM resonators in the same quartz substrate in an array configuration.

Through the years, several MQCMs have been developed for advanced sensing applications, including electronic nose, electronic tongue, and biosensor arrays [8,9,10,11,12,13,14]. The use of MQCM technology leads to relevant benefits including multi-analysis capabilities, lower cost per sensor unit, less sample/reagent consumption, faster sensing response, and shorter detection assay time. All of these advantages arise from having a large number of independent sensing spots in a smaller surface [7,13]. If high sensitivity and multi-analysis detection are simultaneously required, a feasible solution goes through a system based on a monolithic array made up of high fundamental frequency QCM (HFF-QCM) devices. The HFF-QCM principle of operation [15,16] is based on the reduction of the plate thickness of a classical QCM, which increases its resonance frequency. According to Sauerbrey´s relation [3], mass sensitivity is directly proportional to the square of its resonance frequency. HFF-QCM devices have been applied both in gas [17,18,19] and liquid media [20]. Improvements in the LoD (limit of detection) of around two orders of magnitude with respect to classical QCM have been reported using this acoustic sensor technology in immunoassays [21].

Furthermore, the sensing surface of HFF-QCM devices must be reduced with increasing frequency [22], leading thus to devices with small footprints that are easier to be integrated in an array. The main drawbacks of HFF-QCM concept are related to lower mechanical stability and difficult handling [23]. A suitable “inverted mesa” design [20,24,25] can be used to overcome these issues by thinning only a small area of the sensor, while the surroundings of the device provide a supporting frame with mechanical strength.

Other acoustic technologies e.g., surface acoustic wave (SAW) or thin film bulk acoustic resonators (FBAR), have the capability to be designed in an array configuration as well. From these technologies, SAW devices and in particular Love-mode acoustic sensors are typically used in liquid medium applications because of their expected higher sensitivity [26]. However, a similar limit of detection has been obtained using HFF-QCM sensors for the same application and under the same experimental and instrumental conditions [27]. The size of a Love wave sensor is bigger than the corresponding HFF-QCM device for the same substrate material and the same operation frequency. Furthermore, the fabrication of Love wave sensors is more complicated and expensive mainly due to the more complex geometry and the necessity of implementing inter-digital transducers (IDTs).

For the reasons exposed above, HFF-QCM sensors based on inverted mesa technology have been selected to constitute the “basic building unit” of a novel acoustic wave array. Obviously, the complexity of the design of HFF-QCM devices increases when a number of HFF-QCM sensors must be integrated together on the same substrate, and in a reduced area, in an array configuration. In this case, the sensor’s basic design requirements include: small size, high quality factor (considering the high operation frequency) and to avoid or reduce as much as possible the presence of inharmonic modes in the vicinity of the fundamental mode.

On the other hand, a minimum thickness of the electrodes is necessary in order to assure good electrical conductivity and produce the ‘energy trapping’ effect [28,29]. This effect confines the thickness shear vibrations under the electrode area, thus improving the quality factor of the sensor and reducing the interferences among resonators in an array configuration [30,31]. Moreover, the combination of an excessive thick electrode together with a large electrode area could lead to the presence of spurious overtones [22]. Usually, the ‘plate-back’ equation is used in common quartz crystal resonator design (5–10 MHz) to properly determine the optimal electrode geometry and thus avoid the presence of these unwanted inharmonic modes [22]. This equation provides very small thicknesses when used for high frequency sensor design (150 MHz), which would lead to low conductivity issues in the sensor electrodes. For example, for a square 150 MHz resonator, with a lateral dimension of 0.55 mm, a maximum thickness of 0.75 nm of gold electrode would be required to avoid the presence of spurious modes according to the ‘plate-back’ equation. Thus, it is necessary to find a compromise solution to obtain a good electrical conductivity, a proper ‘energy trapping’ effect, and a sufficient separation between the fundamental thickness shear mode and the first spurious mode in order to minimize its interference during the operation of the sensor in liquid medium. In this sense, Equation (1) proposed by Sheahan [32] is suitable to estimate the resonance frequency of the different vibration modes of the device described in this work:

(1) $$\omega_{1mn}\;=\;\left(\pi /\sqrt{\rho }\right)\sqrt{C_{66}/l_{y}^{2}\;+\;m^{2}\left(C_{11}\;+\;\pi^{2}C_{66}/12\right)/l_{x}^{2}\;+\;n^{2}C_{55}/l_{z}^{2}},$$

where $\omega_{1mn}$ is the angular frequency of the different *m* and *n* modes, $C_{66}$, $C_{55}$, $C_{11}$ are the corresponding AT-cut quartz elastic constants, ρ is the quartz density, $l_{y}$ the dimension in the thickness direction, and $l_{x}$ and $l_{z}$ are the lateral dimensions of the resonator. The presence of inharmonic modes in the proximity of the fundamental mode can be minimized by reducing these lateral dimensions. Simulation tools based on numerical methods are commonly used to support the design stage because of its complexity.

Additionally, HFF-QCM sensors are usually employed under flow conditions. In such cases, a suitable flow measurement cell is a basic requirement. Different designs have been proposed in the literature for gas [33] and liquid [23] flows. In the concrete case of bio-sensing applications, most of the measurements are carried out in liquid medium. Then, the cell must confine the liquid over the sensor’s surface while isolating the electrical connections [23]. The key design prerequisites are: low distortion of the original response of the sensor, and reasonable baseline stability. In the case of HFF-QCM devices and due to their special fragility and small sensing area, a very small flow chamber volume and a design approach that assures sensor mechanical integrity and easy replacement are critical additional requirements. Finally, the biocompatibility and chemical resistance of the materials of the flow module that are in contact with the liquid medium must be taken into account.

As a first step to developing a MQCM device, a single HFF-QCM sensor operating a 150 MHz has been designed and manufactured. A flow cell module has also been designed and manufactured to test the sensor in bio-sensing applications. Finally, the sensor and the cell have been validated through a simple molecular interaction experiment using Protein A and IgG antibody.

## 2. Materials and Methods

### 2.1. Sensor Numerical Model

A numerical model of the sensor based on the finite element method (FEM) has been developed to simulate and optimize the electromechanical response of the acoustic wave microsensor prior to fabrication. A commercial software package (ANSYS, Canonsburg, PA, USA) has been used. In order to consider the piezoelectric nature of the AT-cut quartz wafer used as a substrate of the sensor, a multi-physics model has been built. Electric field and mechanical stress/strain have been calculated in the model. Degrees of freedom (DOF) of the model nodes include temperature, electric potential, and 3D displacement. Values for the physical properties of AT-cut quartz, which are described elsewhere [22,31,34], are listed in Appendix A. These properties are obtained starting from those of the ‘mother’ quartz through rotation transformation formulas [35].

A sensitivity analysis has been carried out to study the performance of different finite elements shapes and mesh configurations. A minimum number of elements is needed in the thickness direction to properly capture the characteristics of the resonance; 10 element layers have been found that model the thickness shear mode with suitable resolution at a reasonable computational cost (time and memory). ANSYS Element ‘SOLID226’ has been chosen to model the device. This is a coupled element, and its nodes have four degrees of freedom: mechanical displacements in the *X*-, *Y*-, and *Z*-axes; and electric potential. Therefore, it is suitable to model problems where the electric and mechanical fields are interacting.

The excitation voltage applied to the top electrode has been chosen to be 0.5 V, while a boundary condition of 0 V has been applied to the bottom electrode. Damping factor has been set to 5 × 10^−5^.

The ‘energy trapping’ effect has been modeled using an indirect method following the Mindlin thin plate theory. According to this theory, the contribution of the electrodes to the sensor response would be equivalent to a proper increment of the density of the quartz crystal in the electroded regions. This increment is called ‘mass loading’, and the effective density predicted by this theory is described elsewhere [31,36,37].

**Figure 1:**
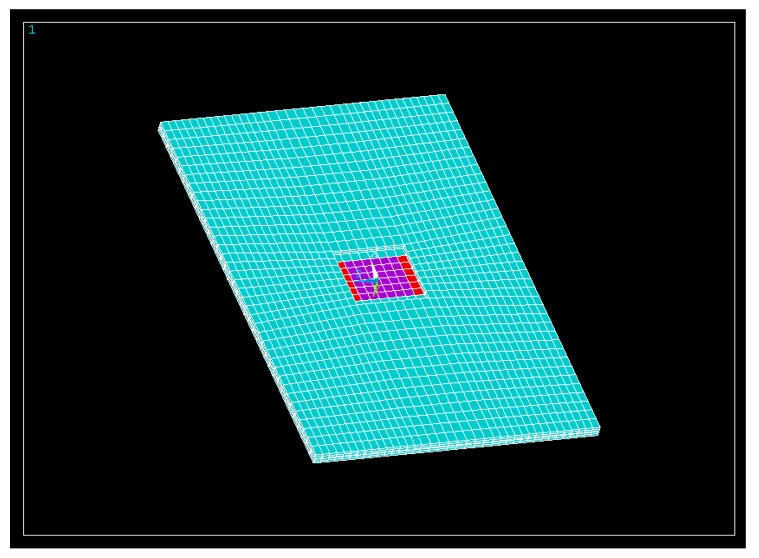
Numerical model of the AWS high fundamental frequency quartz crystal microbalance (HFF-QCM) 150 MHz sensor.

In Figure 1, a numerical model of the 150 MHz resonator is shown. In blue, the bare AT-cut quartz region is represented. The regions where only the top or bottom electrodes appear are indicated in red. Finally, the regions where the top and bottom electrodes overlap are depicted in purple.

### 2.2. Chemicals

Phosphate-buffered saline (PBS) tablets (dissolved in deionized water: 0.01 M phosphate buffer, 0.0027 M potassium chloride and 0.137 M sodium chloride, pH 7.4, at 25 °C), Protein A from Staphylococcus aureus, and IgG from human serum were purchased from Sigma-Aldrich (Sigma-Aldrich Química S.L.U., Madrid, Spain).

### 2.3. Instruments and Devices

**Figure 2:**
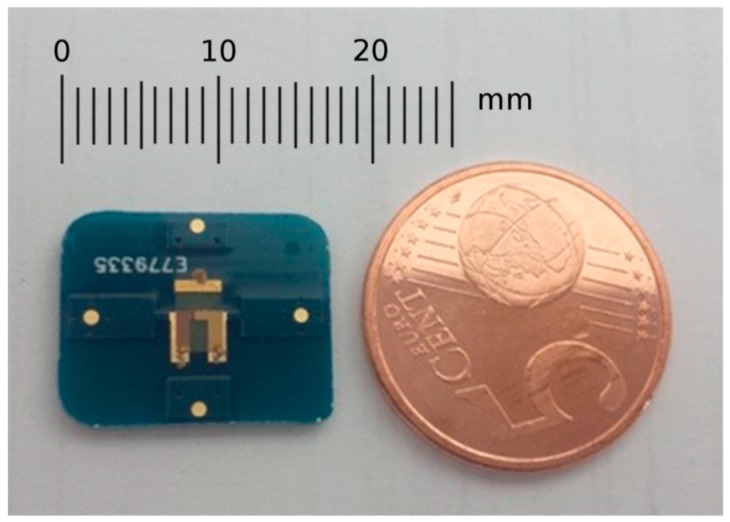
HFF-QCM with a fundamental resonance frequency of 150 MHz manufactured by Advanced Wave Sensors S. L.

The resonator based on inverted mesa technology has been manufactured by using wet etching and metal sputtering technologies [16]. The final sensor includes the resonator mounted over a custom designed PCB (printed circuit board) in order to improve the device handling and robustness. PCB is rectangular, with dimensions 17.5 mm × 14 mm. Its thickness is 1.55 mm. In Figure 2, a picture of the sensor (AWS CW 150 MHz, Advanced Wave Sensors S. L., Paterna, Valencia, Spain) is shown.

### 2.4. Electrical Characterization of the Sensor

The real and imaginary parts of the electrical admittance spectrum of the sensor have been measured using a network analyzer DG8SAQ VNWA 3 (SDR-Kits, Melksham, Wiltshire, UK). The AWS A20 platform (Advanced Wave Sensors S. L.) has been used to characterize the frequency and damping shifts of the sensor response, in real time, during the experiments carried out in flow conditions. The AWS F20 platform (Advanced Wave Sensors S. L.) has been used to generate a uniform flow through the sensor cell. The AWSuite software interface (Advanced Wave Sensors S. L.) has been used to control both platforms during the experiments and to register and process the acquired data.

### 2.5. Flow Cell

A flow cell has been designed and manufactured. The sensor cell includes two main assemblies. The sensor is placed on the bottom assembly that, in turn, acts as the electrical interface between the flow cell and the measurement system. The body is made of aluminum (Aluminum 5083 H111, Broncesval S. A., Paterna, Valencia, Spain) and includes four gold spring contacts that support the sensor, assuring the electrical contact with the external connectors of the cell. These spring contacts allow the sensor to be maintained in a floating-like state, which controls the overpressures on the sensor, thus assuring its integrity and improving its performance in terms of noise and stability. The electrical connections of the cell have been specifically designed to be compatible with the AWS A20 platform connection diagram.

**Figure 3:**
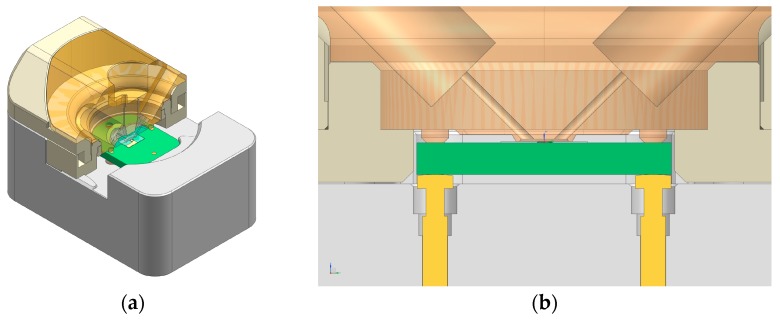
(**a**) Cell 3D view with a cross section detail of the upper part and (**b**) a section detail of the complete cell assembly where the flow chamber is represented in brown, the sensor printed circuit board (PCB) in green, and gold contacts in yellow.

**Figure 4:**
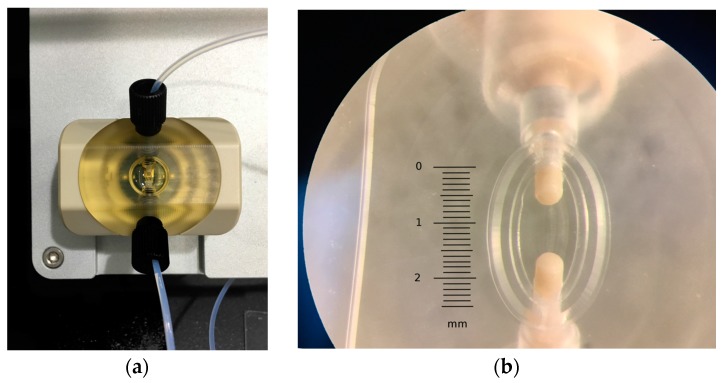
Pictures of (**a**) the measurement cell once assembled in the A20 platform, and (**b**) detailed view of the OSTEMER gasket with the flow chamber, including the flow terminal connections for the fittings.

The upper assembly includes a transparent central core fabricated in PSU (Polysulfone, Ensinger S. A., La Llagosta, Barcelona, Spain), where a custom OESTEMER (OSTEMER Flex, Mercene Labs AB., Stockholm, Sweden) gasket has been embedded to create a flow chamber of 0.53 μL over the sensor. The fluidic channels are built in this structure with a standard fitting termination in order to be easily connected to the external tubing. The PSU core is mechanically attached to a ring-shaped PEEK (Polyether-ether-ketone, Ensinger S. A., La Llagosta, Barcelona, Spain) part that provides a quick and user-friendly lock of the cell. This design avoids using screws, thus assuring a constant pressure over the sensor and a high repetitiveness in its response after cyclic open–close procedures. All the parts of the cell have been fabricated in a vertical CNC (computer numerical control) machining center (Chevalier 1418VMC-Plus, Falcon Machine Tools Co. Ltd., Chang Hua, Taiwan). In Figure 3a, the 3D design of the cell and a section detail of the upper part assembly is shown. In Figure 3b, a section detail of the complete cell assembly is represented where the flow chamber is represented in brown, the sensor PCB in green, and gold contacts in yellow. In Figure 4a,b, the upper part of the cell once assembled in the platform and a detailed view of the OSTEMER gasket with the flow chamber, including the flow terminal connections for the fittings, are respectively shown.

### 2.6. Sensor Cleaning/Preparation

The sensor surface was pretreated for 10 min with UV/ozone cleaner (BioForce Nanosciences Inc., Chicago, IL, USA), rinsed with 99% ethanol, rinsed with bi-distilled water, dried with Nitrogen gas, and treated for a second time with UV/ozone for 10 min.

### 2.7. Protein A Adsorption over Gold Sensor Surface and IgG Binding to the Protein A

The cleaned sensor was placed in the cell and exposed to PBS at a flow rate of 50 μL/min until baseline stabilization. 250 μL of Protein A at a concentration of 50 μg/mL in PBS flowed over the exposed sensor surface at the above flow rate. Different solutions of IgG from human serum at a concentration range of 0.01–10 μg/mL in PBS were added, followed by buffer (PBS) rinse, for 5 min each at the above flow rate.

## 3. Results and Discussion

### 3.1. Simulation

As a result of the design process, an AT-cut quartz wafer with a thickness of 66 microns and lateral dimensions of 5 mm × 7 mm has been selected to be the sensor substrate. This thickness assures enough mechanical robustness, while the lateral dimensions are large enough to allow easy handling of the device, but small enough to be cost-optimal from a manufacturing point of view. A square geometry has been proposed for the sensor to simplify the design of the fluidics and the routing of the electric contacts in the future array. Then, a combination of a gold layer of 60 nm in thickness and an initial Chromium adhesion layer of 5 nm in thickness have been selected to implement the sensor electrode and achieve a good electrical conductivity. This value is lower than the thicknesses used by other authors [15,16], with the objective of avoiding unwanted spurious modes close to the fundamental mode. Later, a squared 0.762 × 0.762 mm^2^ inverted mesa region of 10 microns’ thickness has been built in the center of the substrate. Applying the Sauerbrey approximation, it is possible to estimate that 10 microns of inverted mesa thickness in combination with an Au 60 nm/Cr 5 nm electrode produces a fundamental resonance frequency near the objective frequency of 150 MHz. Finally, applying the Sheahan Equation (1) constrained by a minimum frequency spacing of 400 kHz between the fundamental mode and the first inharmonic, a layout of 0.55 mm × 0.55 mm has been selected to be placed in the center of the inverted-mesa region.

**Figure 5:**
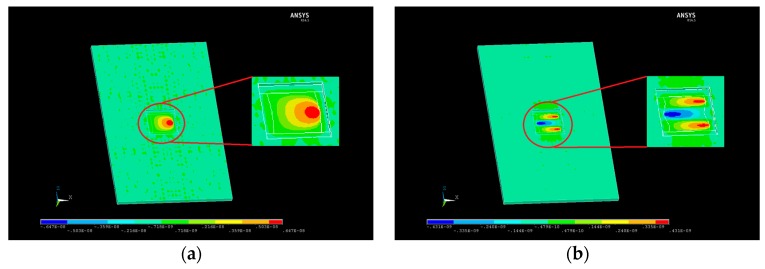
*X*-axis displacement patterns for the (**a**) fundamental thickness shear mode; and (**b**) first inharmonic mode.

Simulations carried out using a FEM model defined by the geometry described in the former paragraph predicted a fundamental resonant frequency of 151 MHz, and a frequency spacing of 430 kHz between the fundamental mode and the first inharmonic. This spacing in frequency is large enough to avoid disturbances while the sensor is operating in liquid medium. The *X*-axis components of displacement in both modes are shown in Figure 5. A maximum displacement in the X-axis of 6.4 nm occurs at the surface of the electroded area when the fundamental mode is excited.

Mechanical energy in the sensor while the fundamental mode is being excited has been calculated through the results provided by the numerical model. Simulation results indicate that 99.992% of this energy is confined in the electroded region, while the rest is distributed all over the AT-quartz substrate, as predicted by ‘energy trapping’ theory [28,29]. Thus, a very low crosstalk level should be expected when this sensor design is employed to implement an array of acoustic wave resonators.

### 3.2. Sensor Characterization

**Figure 6:**
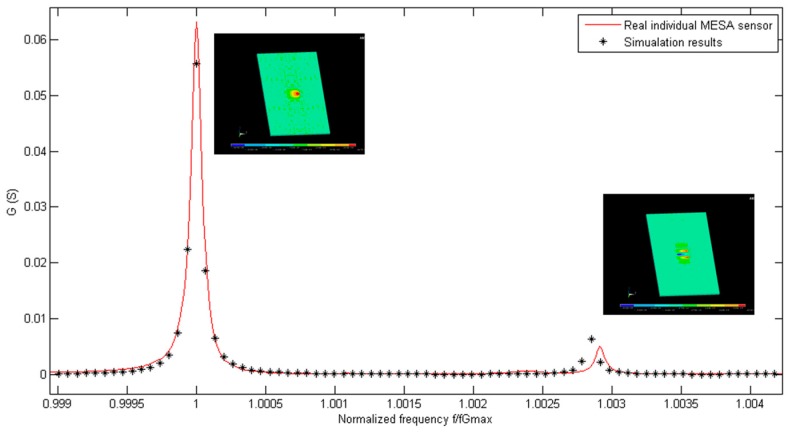
Comparison of the real part of the electrical admittance (G) measured in air of a manufactured AWS HFF-QCM sensor, and the response provided by the numerical model.

**Figure 7:**
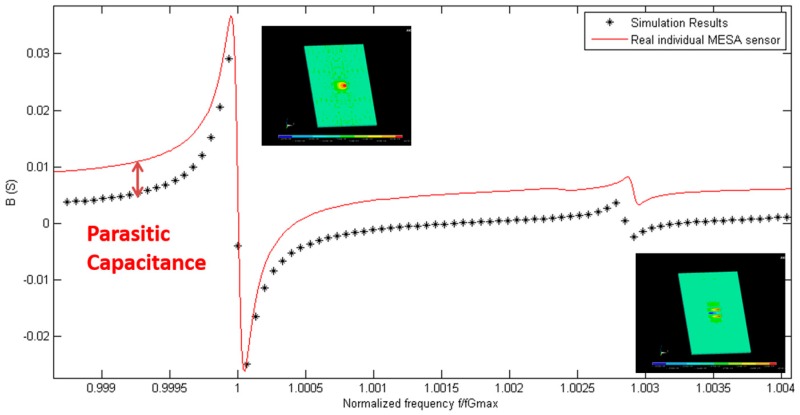
Comparison of the imaginary part of the electrical admittance (B) measured in air of the manufactured AWS HFF-QCM sensor, and the response provided by the numerical model.

The electrical admittance spectrum of the implemented sensor has been acquired. The fundamental and the first inharmonic modes have been included in the frequency range of interest. The average fundamental frequency of a batch of 10 manufactured sensors in the air is 149,811,740 Hz, with a standard maximum deviation smaller than ±250 kHz. The experimental spectrum of the sensor admittance measured in air has been compared with the admittance calculated in the simulations. In Figure 6, a real part of the electrical admittance of the 150 MHz resonator is shown. The black points represent the simulation points at different excitation frequencies, while the red line represents the measured response by using a network analyzer. In Figure 7, the same information is included for the imaginary part of the admittance spectrum of the resonator. The frequency axis has been normalized in both figures for a better comparison of the vibration modes. By doing so, the tolerances in the fabrication process that can slightly affect the thickness of the inverted mesa section, and thus the experimental resonant frequency, are considered. Results show a very good agreement between experimental characterization and numerical predictions. This fact supports the different boundary conditions, meshing options, and assumptions e.g., “mass loading” theory [31,36,37] considered during the development of the numerical model. The main difference between the experimental and numerical data is the offset found in the imaginary part of the admittance. This effect is due to the parasitic capacitance present in the real sensor, and is not taken into account in the model. This is explained by the unavoidable parasitic capacitance, in parallel with the sensor, introduced by the sensor cell. Nevertheless, this fact does not invalidate the model, and it will be considered in the following design steps.

The quality factor (Q) of the sensor has been characterized in air and bi-distilled water conditions using the network analyzer described in Section 2. A quality factor of 9500 has been measured in air, while a Q of 600 has been obtained in water.

Q factors of other HFF-QCM sensors have been previously reported in the literature. Lin et al. [38] reported Q factors of 38,300 and 1170 in air and water, respectively, for a 30 MHz HFF-QCM, and Rabe et al. [25] informed about Q factors of 50,000 and 1110 for their 50 MHz HFF-QCM. As it is well-known, the quality factor of the quartz resonator is inversely proportional to its resonant frequency [39], so it is expected that the 150 MHz resonator presented in this work has a lower Q factor when compared with other resonators with lower resonant frequencies. It is worth noting that while the Q factor in air of our 150 MHz resonator is between four and five times lower that those presented by Lin et al. and Rabe et al., the Q factor in water is only reduced by a factor of two. This fact can be explained if the penetration depth of the acoustic wave in the liquid medium is considered [4,40]. As the resonance frequency becomes higher, the penetration depth decreases together with the energy losses due to the liquid.

### 3.3. Sensor Validation

**Figure 8:**
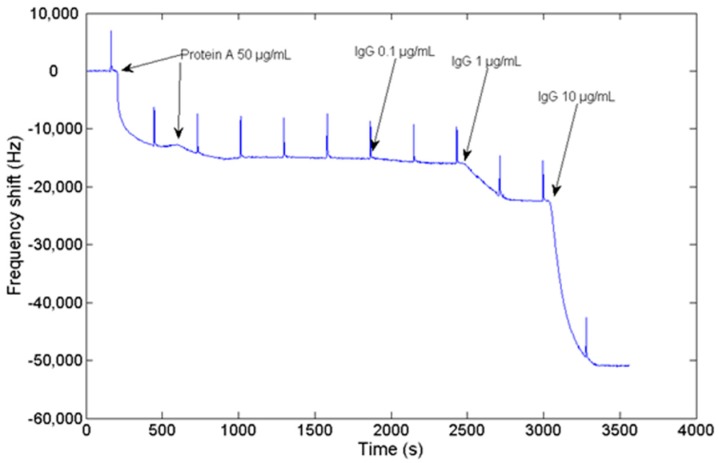
Frequency shift measured during the adsorption of Protein A over the surface of the sensor, and the subsequent injection of 0.1 μg/mL, 1 μg/mL and 10 μg/mL of IgG in the sensor cell.

**Figure 9:**
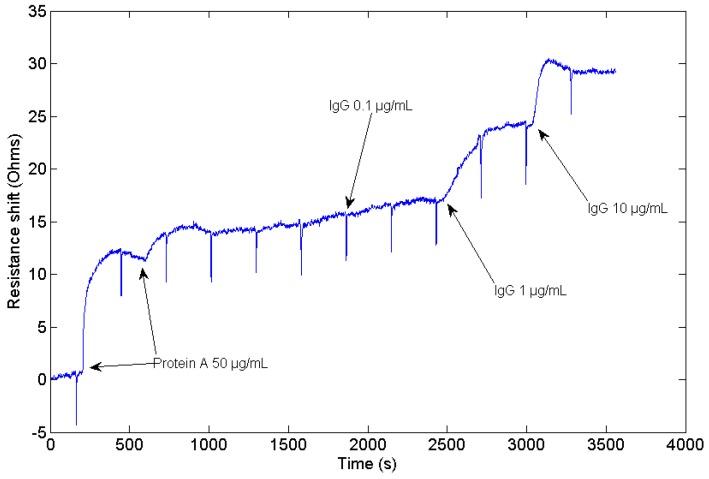
Resistance shift measured during the adsorption of Protein A over the surface of the sensor, and the subsequent injection of 0.1 μg/mL, 1 μg/mL and 10 μg/mL of IgG in the sensor cell.

An experiment consisting of Protein A adsorption to the sensor gold surface and a subsequent IgG binding to the Protein A has been conducted to validate the performance of the HFF-QCM device in bio-sensing applications. The real-time sensor resonant frequency and resistance shifts acquired during the experiment are shown in Figure 8 and Figure 9 respectively. Resistance is commonly used in QCM experiments to evaluate the energy dissipation in the sensor. At the beginning of the experiment, absolute resistance and resonant frequency were 582 Ω and 149,510,403 Hz, respectively. Periodic peaks that can be observed in the graphs are due to the syringe pump used to create the flow over the sensor surface. The syringe must be re-filled every 5 min. During this very short process (2 s), the flow rate over the sensor surface is zero, and the pressure changes. This produces a change in the sensor conditions, and the baseline changes. First, two consecutive injections of protein A (50 μg/mL) have been conducted to make sure that the sensor surface is well covered. In response to the protein A injections, a change both in frequency and resistance is observed. A total negative shift of −9380 Hz in frequency and a positive change in resistance of 10.5 Ω were measured.

Once Protein A has been absorbed over the surface of the sensor, several injections with different concentration of IgG have been carried out. The response of the sensor to injections to 0.1 μg/mL, 1 μg/mL, and 10 μg/mL of IgG provided frequency shifts of −582 Hz, −5585 Hz, and −28,630 Hz, respectively, as depicted in Figure 8. No detectable changes are observed in resistance when 0.1 μg/mL sample is injected. An increase in resistance of 7.5 Ω is detected when 1 μg/mL IgG is injected, while an increment of 4.8 Ω is registered when 10 μg/mL of IgG are injected. Baseline remains stable once the sample volume has passed through the sensor.

Sensor validation results prove that this device can be employed to monitor biomolecular interactions carried out in liquid medium. A stable baseline both in dissipation and in resonant frequency is observed when buffer is passed through the sensor cell. Once Protein A is injected into the flow cell, a negative shift in frequency and a positive shift in resistance are obtained, which is consistent with a typical QCM protein adsorption experiment [41]. The measured frequency shift produced by the combined adsorption of Protein A and the subsequent injection of IgG has a value of −51,000 Hz, as expected, much higher than other experiments described in the literature, which report a frequency shift of around −200 Hz using conventional 9 MHz QCM sensors [42,43]. This fact reveals the enhanced sensitivity of the HFF-QCM device.

## 4. Conclusions

A HFF-QCM sensor based on inverted-mesa technology has been designed to be the ‘basic building unit’ of a future MQCM device. Sensor basic design requirements for this concrete application included small size, good quality factor (considering the high operation frequency), and limitation of the presence of inharmonic overtones in the vicinity of the fundamental mode. Following these specifications, a theoretical study has been carried out to define an initial geometry for the resonator. Then, a numerical FEM model has been developed to estimate the characteristics of such a sensor. A HFF-QCM device based on this design has been implemented and characterized. Comparison between experimental and numerical results showed a very good correlation.

After the implementation and validation of the AWS HFF-QCM sensor of 150 MHz, this sensor is going to be used as the “basic element” of an array. Thus, a novel acoustic wave array will be made up by a matrix of this type of sensors. Of course, it is necessary to design the spacing between resonators in the *X*- and *Z*-axes so that the crosstalk between neighbor resonators is minimized. New numerical FEM models based on the one presented in this paper will be used to find an optimal design solution prior to array fabrication. This work will be presented in forthcoming articles.

## Appendix A

**Table A1:** Values of the piezoelectric, dielectric and elastic constants of the AT-cut Quartz.

Dielectric Matrix $\left[\varepsilon^{S}\right]$	$\left(\begin{array}{ccc} 39.21 & 0 & 0\\ 0 & 39.82 & 0.86\\ 0 & 0.86 & 40.42 \end{array}\right)\cdot 10^{-12}$
Elastic Matrix $\left[c^{E}\right]$	$\left(\begin{array}{cccccc} 86.74 & -8.25 & 27.15 & -3.66 & 0 & 0\\ -8.25 & 129.77 & -7.42 & 5.7 & 0 & 0\\ 27.15 & -7.42 & 102.83 & 9.92 & 0 & 0\\ -3.66 & 5.7 & 9.92 & 38.61 & 0 & 0\\ 0 & 0 & 0 & 0 & 68.81 & 2.53\\ 0 & 0 & 0 & 0 & 2.53 & 29.01 \end{array}\right)\;\cdot 10^{9}$
Piezoelectric Matrix [e]	$\left(\begin{array}{c} 0.171\\ 0\\ 0 \end{array}\;\begin{array}{c} -0.152\\ 0\\ 0 \end{array}\;\begin{array}{c} -0.0187\\ 0\\ 0 \end{array}\;\begin{array}{c} 0\\ 0.108\\ -0.0761 \end{array}\;\begin{array}{c} 0\\ -0.095\\ 0.067 \end{array}\right)$

**Table A2:** Quartz and gold densities.

ρ gold	19,300	kg/m^3^
ρ quartz	2650	kg/m^3^
